# Advances and Clinical Outcomes in Hodgkin Lymphoma in the Era of Novel Therapies

**DOI:** 10.3390/jcm12051928

**Published:** 2023-03-01

**Authors:** Annalisa Paviglianiti, Nicolò Rampi

**Affiliations:** 1 Clinical Hematology Department, Institut Català d’Oncologia-Hospitalet, 08908 Barcelona, Spain; 2 Institut d’Investigació Biomèdica de Bellvitge (IDIBELL), 08908 Barcelona, Spain; 3 Department of Oncology and Hemato-Oncology, University of Milan, 20122 Milan, Italy

Hodgkin lymphoma (HL) is traditionally considered one of the hematological malignancies with the highest rate of cure, ranging from 70 to 90% depending on the disease and patient features. Along with the role of positron emission tomography (PET), therapeutical regimens are progressively shaped by the progressive clinical use of novel targeted agents.

The anti-CD30 antibody-drug conjugate brentuximab vedotin (BV) has completely revolutionized the treatment of refractory/relapsed (R/R) HL [1]. After the results of the AETHERA trial, consolidation with BV before and after autologous stem cell transplantation became the standard of care in high-risk patients [2]. Subsequently, BV use in combination with other drugs was further reported in relapse [3,4] and in frontline settings in advanced-stage disease with encouraging results for both progression-free [5] and overall [6] survival.

Beside BV, checkpoint inhibitors (CPIs) such as nivolumab and pembrolizumab have drastically changed the treatment of HL. CPIs have proven to be very effective at disease control for heavily pretreated patients [7,8] and have a relatively well-tolerated profile. In the NIVAHL trial, at the most recent 3-year follow-up update, a progression-free survival of around 100% was registered when nivolumab was given sequentially or combined with chemotherapy in early-stage unfavorable HL [9]. Similar results were obtained when pembrolizumab was associated with gemcitabine, vinblastine and liposomal doxorubicine in an R/R setting, demonstrating that it is a feasible and well-tolerated bridging therapy in transplant situations [10]. A phase 1–2 study with nivolumab and BV as first salvage therapy was proposed by Advani et al., demonstrating impressive results in a chemo-free setting [11].

As checkpoint inhibitors were originally studied in BV-failure settings in particular, many questions were raised around their early use due to their efficacy. A head-to-head comparison between pembrolizumab and BV was therefore performed, showing the superiority of the former in terms of disease control [12].

The most refractory patients, including those requiring multiple lines of therapy to achieve a response or those relapsing after an autologous hematopoietic cell transplantation (HCT), may achieve long-term survival with allogeneic HCT [13]. For patients who are refractory to chemotherapy and show progression after BV- and CPI-containing regimens, there is no standard of care. Enrollment in clinical trials is advised to achieve disease response prior to allogeneic HCT.

Given the progress of chimeric antigen receptor T-cell (CAR-T) therapy, there are several ongoing trials involving CD30-directed CAR-T in cHL (RELY-30, CHARIOT) [14] and preclinical investigations into bispecific antibodies.

Substantial progress has been made in the last decade in the management of HL and in terms of our knowledge of its biology, but the treatment of R/R disease is still challenging. Through this Special Issue, we hope to offer an overview of the advances and clinical outcomes in the modern era.
